# Heatwaves diminish the survival of a subtidal gastropod through reduction in energy budget and depletion of energy reserves

**DOI:** 10.1038/s41598-017-16341-1

**Published:** 2017-12-15

**Authors:** Jonathan Y. S. Leung, Sean D. Connell, Bayden D. Russell

**Affiliations:** 1 Southern Seas Ecology Laboratories, The Environment Institute, School of Biological Sciences, The University of Adelaide, South Australia, Australia; 2 The Swire Institute of Marine Science and School of Biological Sciences, The University of Hong Kong, Hong Kong SAR, China

## Abstract

Extreme climatic events, such as heatwaves, are predicted to be more prevalent in future due to global climate change. The devastating impacts of heatwaves on the survival of marine organisms may be further intensified by ocean acidification. Here, we tested the hypothesis that prolonged exposure to heatwave temperatures (24 °C, +3 °C summer seawater temperature) would diminish energy budget, body condition and ultimately survival of a subtidal gastropod (*Thalotia conica*) by pushing close to its critical thermal maximum (CT_max_). We also tested whether ocean acidification (*p*CO_2_: 1000 ppm) affects energy budget, CT_max_ and hence survival of this gastropod. Following the 8-week experimental period, mortality was markedly higher at 24 °C irrespective of *p*CO_2_ level, probably attributed to energy deficit (negative scope for growth) and concomitant depletion of energy reserves (reduced organ weight to flesh weight ratio). CT_max_ of *T*. *conica* appeared at 27 °C and was unaffected by ocean acidification. Our findings imply that prolonged exposure to heatwaves can compromise the survival of marine organisms below CT_max_ via disruption in energy homeostasis, which possibly explains their mass mortality in the past heatwave events. Therefore, heatwaves would have more profound effects than ocean acidification on future marine ecosystems.

## Introduction

Since the onset of Industrial Revolution, atmospheric carbon dioxide (*p*CO_2_) concentration has been increasing rapidly and caused observable changes in marine ecosystems. For example, sea surface temperature increased by ~0.76 °C in the last century and is predicted to increase by 1.8–3.5 °C by the end of this century, depending on the CO_2_ emission scenario^1^. The increase in *p*CO_2_ is also leading to ocean acidification (OA), which is expected to have profound repercussions on a variety of marine organisms^2,3^. Indeed, meta-analyses suggest that OA and warming will likely modify community structures and functions of future marine ecosystems^4,5^.

Given the slow rate of change in seawater chemistry, however, growing evidence shows that marine organisms may be able to acclimate to the predicted climatic conditions^6–8^. As such, it has been suggested that extreme climatic events, rather than climatic trends, will lead to more significant changes in future marine ecosystems due to their instant and catastrophic effects^9^. Heatwaves, one of the most devastating extreme climatic events, are of special concern because they can abruptly increase the sea surface temperature along coastlines by ~3 °C on average and persist for more than two months, causing substantial changes in marine ecosystems^10–12^. In view of global warming, it is conjectured that heatwaves will be more frequent and persistent in future^9,13^. Indeed, 71% of coastlines worldwide have already been warmed to some extent, while 38% have experienced anomalously high seawater temperature due to extreme hot events^14^.

Heatwaves can greatly influence the survival and distribution of marine organisms, and hence functions of marine ecosystems^11,12^. Cool-affinity species with low mobility are particularly vulnerable due to their limited ability to escape from thermal stress, while heatwaves favour the proliferation of warm-affinity species^12^. This implies that thermal stress is the key factor causing the mass mortality in the past heatwave events. Therefore, studying thermal tolerance of marine organisms can provide an initial insight into how heatwaves affect their fitness and survival^15^. Nevertheless, thermal tolerance could be modulated by OA through disruption in aerobic performance or energy homeostasis^16,17^, possibly increasing the susceptibility of marine organisms to heatwaves. Indeed, marine organisms are impacted by environmental stressors commonly via reduction in aerobic scope, defined as the excess metabolic energy available after basal metabolic cost is met^18^. As such, studying bioenergetics can shed light on the combined effects of multiple stressors on the performance, fitness and survival of marine organisms^18^. If heatwaves or OA causes energy deficit (i.e. negative energy budget) in the long term, energy reserves would be consumed or even depleted, eventually leading to mortality.

Here, we examined the effects of prolonged exposure to elevated *p*CO_2_ and temperature on the energy budget, body condition and survival of a subtidal grazing gastropod *Thalotia conica*, which is widely distributed in temperate Australia. Given its high abundance, this seagrass-associated gastropod plays a crucial role in energy flow in the seagrass community by regulating the abundance of epiphytes on seagrass leaves^19^, which can enhance the survival of seagrasses^20^. We first assessed the performance of aerobic metabolism of *T*. *conica* along a temperature gradient and determined its critical thermal maximum (CT_max_), at which motor coordination is lost^21^, as a proxy for thermal tolerance. Then, we hypothesized that (1) prolonged exposure (8 weeks) to heatwave temperatures would lead to increased mortality of *T*. *conica* by diminishing its energy budget and body condition, when the temperature is close to CT_max_; and (2) OA would reduce energy budget and thermal tolerance, resulting in greater mortality. Provided prolonged exposure to heatwaves diminishes the survival of this subtidal gastropod through energy depletion even below its thermal tolerance, this mechanism not only helps explain the mass mortality of marine organisms in the past heatwave events, but also suggests that persistent heatwaves are the main driver of the functions of both contemporary and future marine ecosystems.

## Results

### Performance of aerobic metabolism and CT_max_

**Figure 1:**
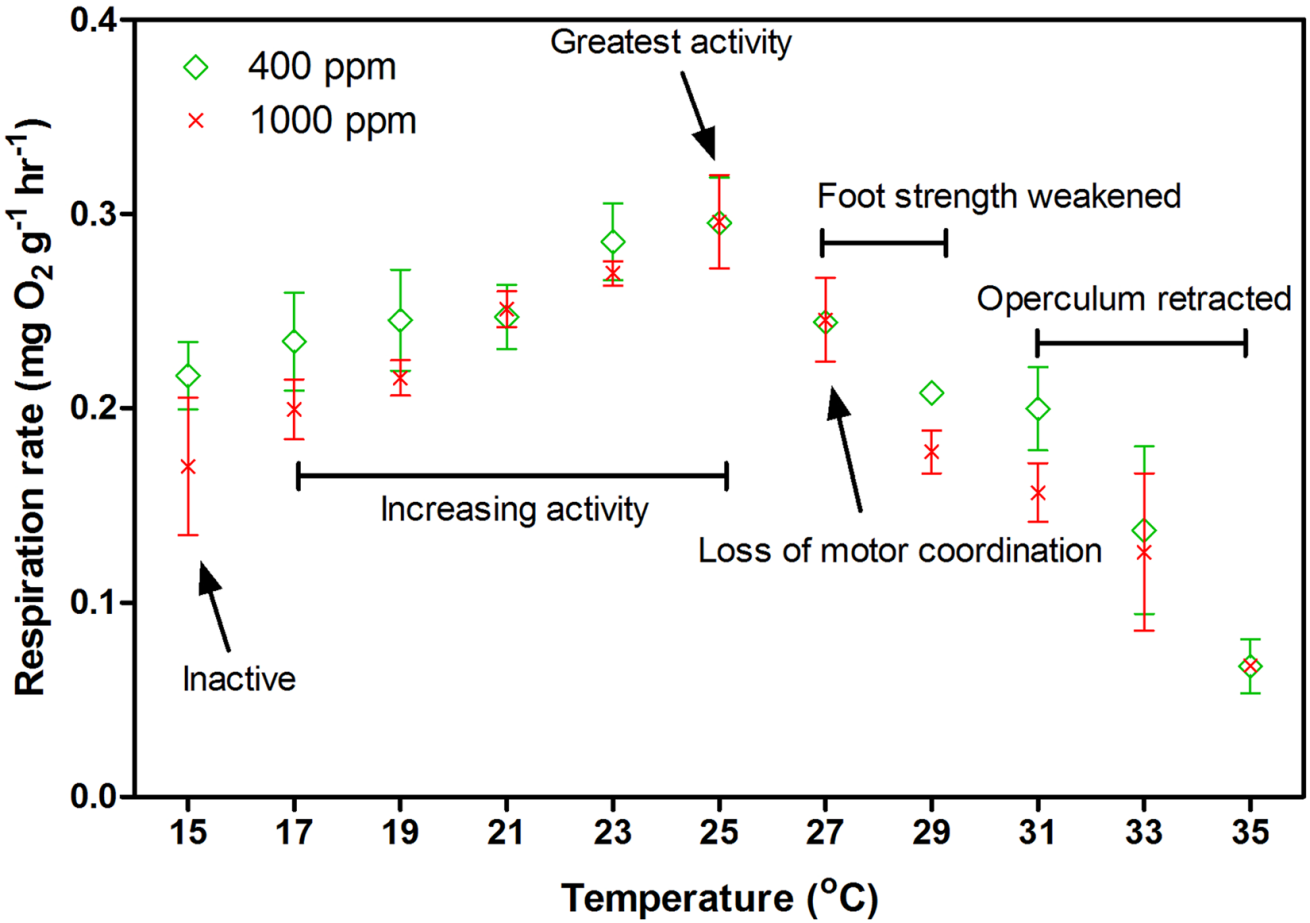
The respiration rate of *Thalotia conica* along an increasing temperature ramp (+2 °C hr^−1^) from 15 °C to 35 °C at different *p*CO_2_ levels (mean ± S.E.; *n* = 3).

The respiration rate of *T*. *conica* gradually increased from 15 °C to 25 °C and then dropped from 25 °C to 35 °C (Fig. 1). The effect of *p*CO_2_ on respiration rate was not discernible, with few exceptions at some temperatures (29 °C and 31 °C). Regarding locomotory behaviour, individuals were inactive at 15 °C, but became gradually more active as temperature increased with the greatest activity at 25 °C. At 27 °C, foot strength was weakened and motor coordination was lost. Beyond 29 °C, the operculum was retracted, indicating acute thermal stress. Based on the locomotory behaviour, the CT_max_ of *T*. *conica* appeared at 27 °C.

### Energy budget

**Figure 2:**
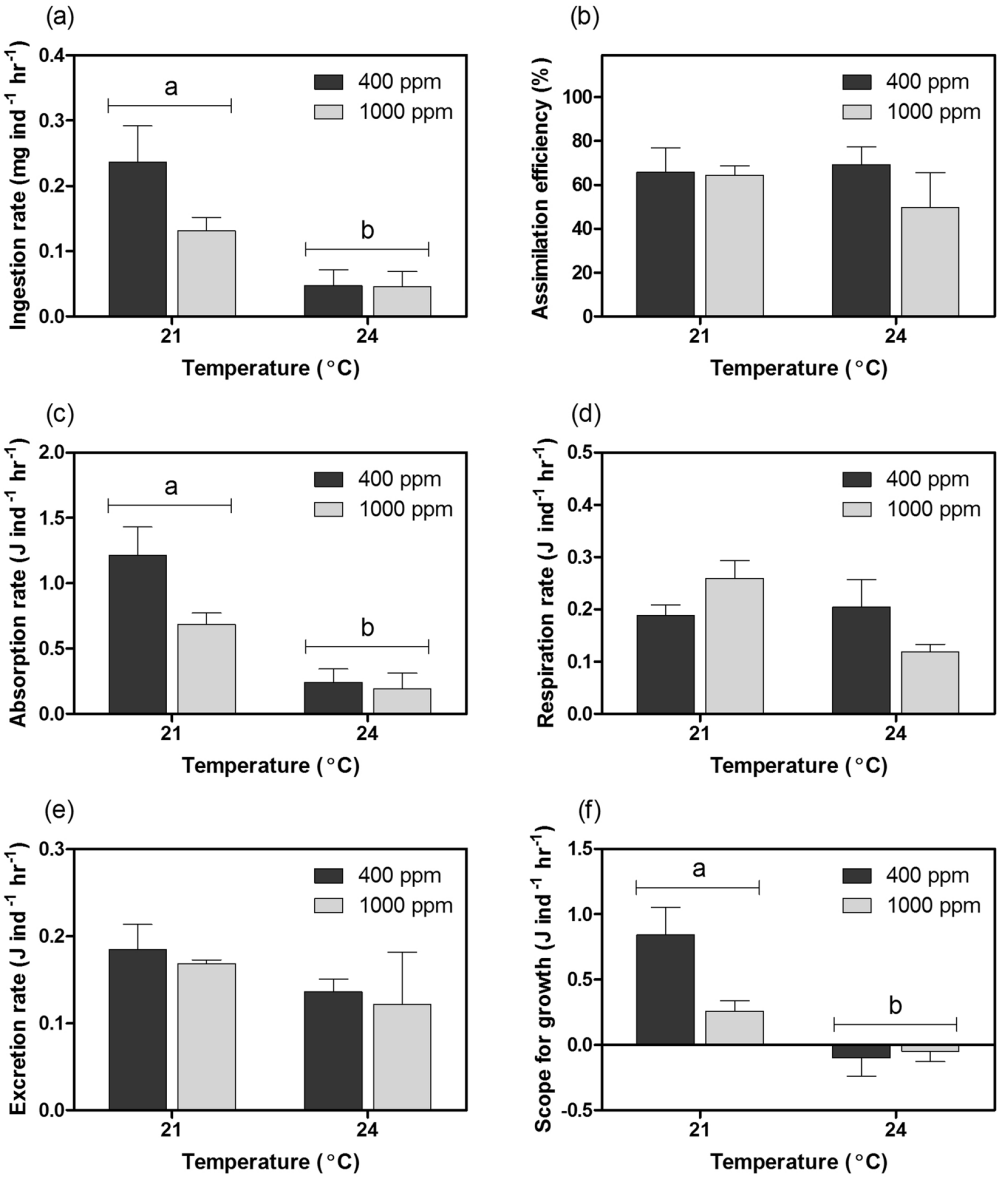
(**a**) Ingestion rate, (**b**) assimilation efficiency, (**c**) absorption rate, (**d**) respiration rate, (**e**) excretion rate and (**f**) scope for growth of *Thalotia conica* under different treatment conditions at the end of the experimental period (mean ± S.E.; *n* = 3). Different letters indicate significant difference between temperature groups (*p* < 0.05).

The ingestion rate, absorption rate and scope for growth of *T*. *conica* at 24 °C were lower than those at 21 °C irrespective of *p*CO_2_ level (Fig. 2a,c and f; Table S1). Assimilation efficiency and excretion rate were not affected by both *p*CO_2_ and temperature (Fig. 2b and e; Table S1). Respiration rate at 24 °C was lower than that at 21 °C when *p*CO_2_ was at 1000 ppm (Fig. 2d; Table S1). Based on the patterns of energy budget parameters, absorption rate was the key factor determining scope for growth. Energy deficit was found at 24 °C, indicated by the negative scope for growth (Fig. 2f).

### Body condition and survival

**Figure 3:**
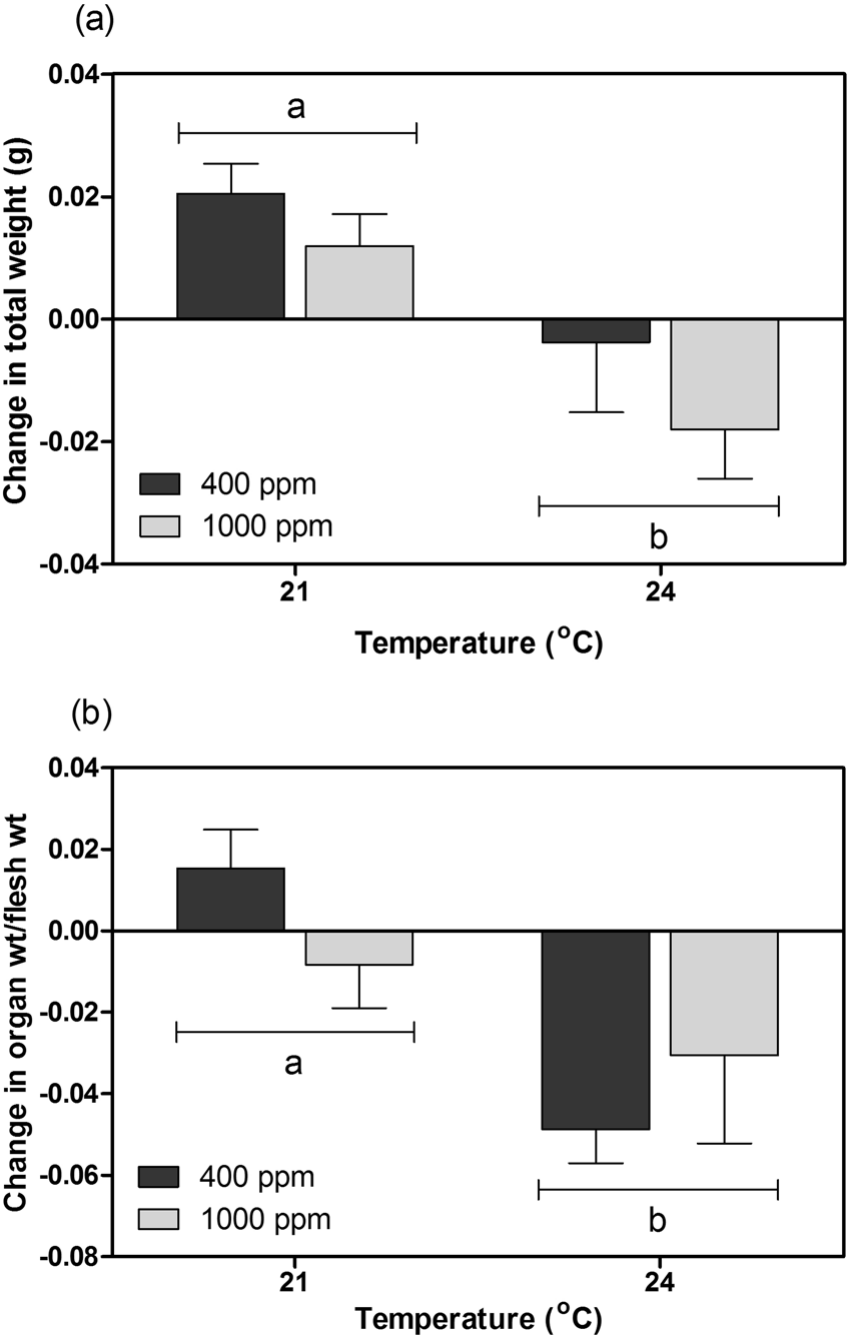
The change in (**a**) total weight and (**b**) organ weight to flesh weight ratio of *Thalotia conica* after the experimental period under different treatment conditions (mean + S.E.; *n* = 22 individuals for 21 °C, 400 ppm; *n* = 21 individuals for 21 °C, 1000 ppm; *n* = 8 individuals for 24 °C, 400 ppm; *n* = 5 individuals for 24 °C, 1000 ppm). Different letters indicate significant difference between temperature groups (*p* < 0.05).

**Figure 4:**
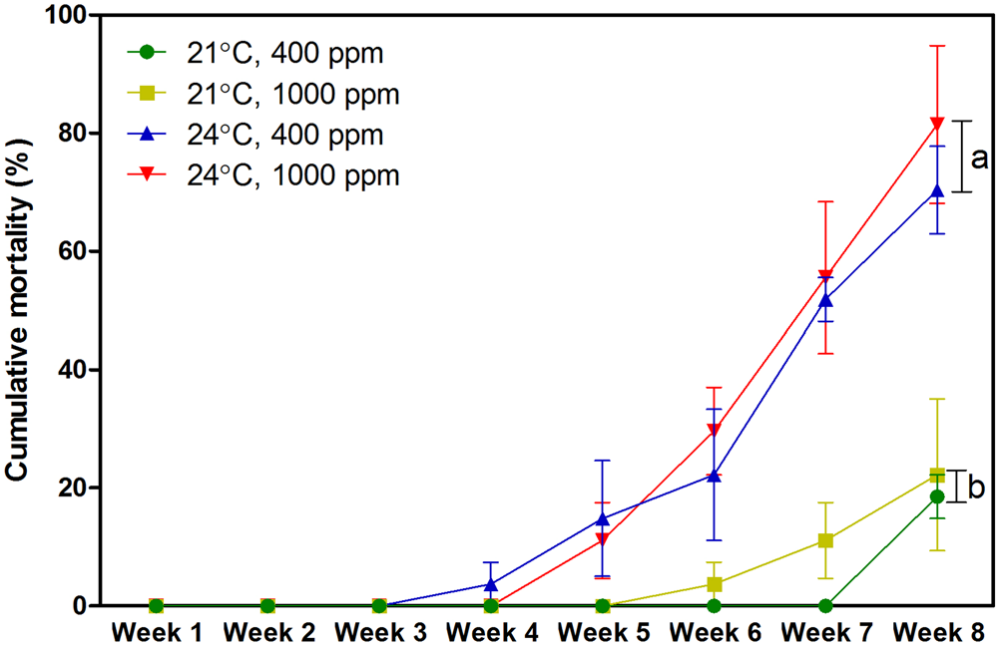
The cumulative mortality of *Thalotia conica* across the experimental period under different treatment conditions (mean ± S.E.; *n* = 3). Different letters indicate significant difference between temperature groups in Week 8 (*p* < 0.05).

Following the 8-week experimental period, the total weight of *T*. *conica* increased at 21 °C, but decreased at 24 °C regardless of *p*CO_2_ level (Fig. 3a; Table S1). Organ weight to flesh weight ratio did not change significantly at 21 °C, but decreased at 24 °C at both *p*CO_2_ levels (Fig. 3b; Table S1), indicating the use of energy reserves. Mortality at 21 °C (~20%) was much lower than that at 24 °C (~70% at 400 ppm; ~80% at 1000 ppm) after the experimental period (Fig. 4; Table S1).

## Discussion

Studying bioenergetics can help decipher the fitness and survival of marine organisms in response to multiple environmental stressors^18^. Contrary to theory^22^, we revealed that OA did not significantly affect the thermal tolerance, energy budget, body condition and survival of a subtidal gastropod, even though the individuals had limited time to acclimate. The slightly reduced energy budget may be elicited by the extra energy required for the maintenance of acid-base balance and digestion efficiency under OA conditions and thus less energy can be allocated for foraging activity^22^, resulting in reduced energy gain by feeding. In contrast, we found that prolonged exposure to heatwave temperature markedly reduces energy budget, body condition and eventually survival. Therefore, heatwaves can probably pose greater adverse consequences than OA on the fitness and survival of marine organisms.

The detrimental effect of heatwaves on organisms can be mediated by reducing their aerobic scope (or excess metabolic energy)^22^. In general, energy demand increases with temperature and thus aerobic respiration needs to be upregulated to produce more metabolic energy and meet the elevated energy demand so that energy homeostasis can be maintained. When metabolic energy produced by aerobic respiration is insufficient to meet the elevated energy demand, aerobic scope will decrease^18,23^, which can be indicated when aerobic respiration starts to decline with increasing temperature^24,25^. As such, the foot strength of *T*. *conica* starts to weaken and its equilibrium is lost at 27 °C (i.e. CT_max_), where the respiration rate can no longer increase with temperature probably due to reduced capacity of ventilatory and circulatory systems to deliver oxygen to foot tissues^23,24^. Consequently, the excess metabolic energy would not be adequate to support normal physiological functions and locomotory activity, and hence survival becomes time-limited, depending on the tolerance of individuals to starvation and thermal stress^24^. To avoid the impact of acute thermal stress, therefore, *T*. *conica* maximized locomotory activity at 25 °C, potentially as an escape behaviour. Since the respiration rate at 24 °C (i.e. heatwave temperature) was very close to the maximum, *T*. *conica* would be under sublethal thermal stress, which can compromise fitness and survival in the long term via disruption in energy homeostasis^23,26^.

As maintaining energy surplus is the key for long-term survival, bioenergetics-based models can be used to examine how environmental stressors affect the fitness and survival of marine organisms by changing their energy budget^18^. In this study, scope for growth was estimated to indicate energy budget, which was subject largely to absorption rate. We found that the heatwave temperature led to reduced absorption rate, which was in turn caused by reduced ingestion rate. The worsened feeding performance is likely attributed to the impact of sublethal thermal stress on aerobic metabolism. Based on the respiration rate along the temperature ramp, *T*. *conica* should have higher respiration rate at 24 °C than at 21 °C. After the experimental period, however, *T*. *conica* could not maintain higher respiration rate at 24 °C, which is likely caused by the reduced oxygen delivery capacity or impaired mitochondrial functions under long-term sublethal thermal stress^23,27,28^. As a result, energy production is suppressed, impairing various physiological functions and activities, including feeding^29,30^. Whether the reduced energy gain by feeding compromises growth and body condition depends on the net energy balance (i.e. scope for growth). We found that scope for growth is negative after the prolonged exposure to heatwave temperatures, indicating energy deficit which probably accounts for the loss of total weight. As shell weight is generally constant, the reduction in total weight plus negative energy budget suggest consumption of energy reserves, which is further substantiated by the reduced body condition (i.e. reduced organ weight to flesh weight ratio), indicating that energy supply was inadequate to support energy demand. Indeed, using energy reserves for basal maintenance becomes inevitable when energy deficit persists for a longer term. For instance, Ivanina *et al*.^31^ demonstrated that prolonged exposure to elevated temperature can cause depletion of energy reserves in bivalves *Crassostrea virginica* and *Mercenaria mercenaria*. Yet, using energy reserves can only provide a temporary relief against environmental stressors and is considered as the last resort for survival. When energy reserves are depleted, physiological processes will be hindered, or even arrested, explaining the rapid increase in the mortality of *T*. *conica* after six weeks of exposure to the heatwave temperature.

To date, heatwaves have received less attention due to their historical rarity in nature, but they can pose destructive impacts on marine ecosystems, especially survival of marine organisms. For example, recent heatwave events have abruptly increased the seawater temperature along coastlines by ~3 °C on average above normal level for more than eight weeks and caused mass mortality of marine organisms with little sign of recovery, possibly culminating in irreversible damage to ecosystem functions (Northwest Mediterranean coast in 1999 and 2003^10,11,32^; Western Australian coast in 2011^12,33^). Although underlying mechanisms (e.g. development of pathogens and modified species interactions) have been postulated to account for the mass mortality^9,12,34^, we suggest that persistent thermal stress is the overarching factor because it can directly impair aerobic metabolism, worsen feeding performance and eventually cause energy depletion^18,23^. Although subtidal organisms, especially in temperate regions, were shown to be resilient to elevated temperature because most of them are living below their thermal tolerance and have high acclimation capacity^35,36^, we showed that the adverse effects of thermal stress can be manifested after prolonged exposure to temperature below thermal tolerance. As many subtidal organisms are not highly mobile or even sessile (e.g. corals, polychaetes, gastropods, bivalves, sea urchins, etc.), they are subject to the instant thermal stress caused by heatwaves and have limited time to respond, ultimately leading to mortality.

In the past decade, a plethora of studies have investigated the potential impacts of OA on future marine ecosystems. Despite the merits of these studies, it is still difficult and challenging to accurately predict the impacts of OA in view of the inconsistent results and complexity of marine ecosystems^4,8,37,38^. Given the slow rate of change in seawater chemistry, the acclimation capacity of marine organisms will further make the consequences of future marine ecosystems unpredictable. In contrast, extreme climatic events, such as heatwaves, can remarkably modify the functions of marine ecosystems in a relatively short period of time^11,12^. In view of the dominance and role of *T*. *conica*, our findings imply that the ecological functions of seagrass community would be substantially altered by heatwaves, whereas OA poses negligible effects in the near future^39,40^.

Owing to global climate change, the frequency and persistence of heatwaves are predicted to increase in future, possibly jeopardizing the survival of marine organisms and modifying the functions of marine ecosystems. We revealed that prolonged exposure to heatwaves significantly diminishes the survival of a subtidal gastropod through reduction in energy budget and depletion of energy reserves, which are manifested at temperature below its thermal tolerance. This bioenergetics-based mechanism could help explain the mass mortality of marine organisms in past heatwave events and suggest that many subtidal organisms in temperate regions are vulnerable to persistent thermal stress, which would be the major driver affecting the integrity of both contemporary and future marine ecosystems.

## Methods

### Collection of gastropods and rearing conditions

Adult *Thalotia conica* were collected in summer from the subtidal region (0.5 to 4 m depth) at Wirrina Cove, South Australia (35°29′S, 138°15′E), where the seawater pH and temperature were similar to our laboratory control conditions at ~8.10 and 21 °C, respectively. Individuals were temporarily kept in a plastic holding aquarium (1 m × 40 cm × 30 cm) filled with natural seawater and allowed to acclimate under ambient conditions (pH: 8.10 ± 0.10, temperature: 21 ± 1 °C, salinity: 35 ± 1 psu and dissolved oxygen concentration: 6.9 ± 0.2 mg O_2_ L^−1^) with natural day/night cycles for two weeks prior to experimentation. Food was provided as epiphytes on rocks from the collection site. Seawater (ca. 50%) was replaced weekly.

### Performance of aerobic metabolism and CT_max_ of gastropods

The performance of aerobic metabolism of *T*. *conica* was assessed by measuring respiration rate from 15 °C to 35 °C with an increasing temperature ramp of 2 °C h^−1^ ^25^, whereas the locomotory behaviour was observed to estimate CT_max_, indicated by the loss of motor coordination as the end point^21,36^. Prior to experimentation, individuals were transferred into plastic holding aquaria for a 2-day acclimation period at lower temperature. To avoid the shock effect due to a sudden decrease in temperature, the aquaria were submerged in a water bath and the seawater temperature in the aquaria was reduced from 21 °C to 15 °C at a decreasing rate of 3 °C day^−1^ using heater/chiller units (TC60, TECO, Italy). After the 2-day acclimation period, two individuals (shell height: ~11 mm) were transferred into a 73 mL airtight chamber filled with experimental seawater (i.e. *p*CO_2_ and temperature manipulated) at 15 °C and allowed to rest for one hour to minimize the effect of handling stress (*n* = 3 replicate chambers per *p*CO_2_ level, see section below for manipulation of *p*CO_2_ level). After the 1-hour resting period, the chamber seawater was fully replaced with oxygen-saturated experimental seawater with initial dissolved oxygen concentration measured using an automatic temperature compensation oxygen optode (LDO101, HACH, USA), which was calibrated at room temperature (~23 °C) following the manufacturer’s instructions. The chamber was then closed and put into a water bath at the target temperature to maintain the temperature of chamber seawater. After one hour, the chamber was opened to measure the final dissolved oxygen concentration, which was above 5.2 mg O_2_ L^−1^ for all samples. Then, the chamber seawater was fully replaced with oxygen-saturated experimental seawater at the next temperature level (i.e. +2 °C) immediately. The procedure for measuring respiration rate was repeated and locomotory behaviour was observed using the same individuals until measurement for the last temperature level (i.e. 35 °C) was completed. Blank samples without individuals were used to correct the background change in dissolved oxygen concentration. Respiration rate was expressed as mg O_2_ g^−1^ (flesh weight) h^−1^, where flesh weight was obtained after dissection.

### Experimental setup for exposure to elevated pCO_2_ and temperature

Individuals of *T*. *conica* (shell height: 11.0 ± 1.30 mm, mean ± S.D.) were transferred into plastic aquaria (30 cm × 20 cm × 18 cm; *n* = 9 individuals per aquarium), which contained 5 L natural seawater, *Ulva* sp. and rocks from the collection site. They were then exposed to one of the crossed combinations of *p*CO_2_ (400 ppm vs. 1000 ppm) and temperature (21 °C vs. 24 °C) for eight weeks (*n* = 3 replicate aquaria per treatment). The “ambient” temperature (21 °C) is the average seawater temperature at the collection site in summer (average annual range: ~14 °C–22 °C), while the elevated temperature (+3 °C) simulates the average increase in seawater temperature based on the past heatwave events^11,12^. The desired seawater temperatures were achieved by submerging the aquaria in water baths maintained by heater/chiller units (TC60, TECO, Italy). The elevated *p*CO_2_ level, which simulates the predicted RCP8.5 scenario for the year 2100^1^, was maintained by aerating the seawater with CO_2_-enriched atmospheric air using a gas mixer (Pegas 4000 MF, Columbus Instruments, USA). Food was provided as epiphytes on rocks from the collection site and replenished before totally consumed. Seawater (ca. 50%) was renewed weekly to prevent accumulation of metabolic waste. The seawater carbonate chemistry parameters throughout the experimental period are shown in Table S2.

### Energy budget of gastropods

To estimate the effect of prolonged exposure to elevated *p*CO_2_ and temperature on energy budget, scope for growth was measured at the end of the experimental period (i.e. Week 8), where respiration rate, absorption rate and excretion rate were determined^41^. To measure respiration rate, two individuals from each aquarium were transferred into a 73 mL airtight chamber filled with seawater adjusted to their respective treatment conditions, and allowed to rest for one hour (*n* = 3 replicate chambers per treatment). The experimental procedures for measuring respiration rate are described in the section above. To quantify absorption rate (i.e. energy gain from the ingested food in a given period of time), two individuals which had been starved for one day were allowed to consume the epiphytes on rocks in their aquarium for one hour under their respective treatment conditions (*n* = 3 replicate aquaria per treatment). The initial and final total weights of each individual were measured by an electronic balance to the nearest 0.0001 g after blotting and removing the seawater inside the shell by gently tapping the operculum to obtain the fresh weight of epiphytes consumed^8^. The water content of epiphytes was determined by oven-drying so that ingestion rate was expressed as dry weight of epiphytes consumed per individual per hour. Then, the two individuals from the same feeding trial were put into a clean container filled with 200 mL natural seawater maintained under their respective treatment conditions. After four hours, the faeces in the container were collected, rinsed with deionized water and dried on a pre-weighed aluminium foil to obtain the dry weight of faeces. The ash-free dry weights of epiphytes and faeces were determined by weight loss on ignition at 550 °C in a muffle furnace for six hours so that assimilation efficiency was calculated according to equation (1) [ref.^42^]:

1 $$AE( \% )=\frac{F^{\prime} -E^{\prime} }{(1-E^{\prime} )\,(F^{\prime} )}\times 100$$

where *AE* is the assimilation efficiency; *F*′ is the ash-free dry weight to dry weight ratio of food; *E*′ is the ash-free dry weight to dry weight ratio of faeces.

Absorption rate is calculated by multiplying ingestion rate by assimilation efficiency, where the ingestion rate is converted into energy equivalent by the calorific value of epiphytes (8227 J g^−1^), determined by bomb calorimeter (C2000 Basic, IKA, Germany). To quantify excretion rate, the ammonium concentration of seawater in the container was analysed using a flow injection analyser (QuikChem 8500, Lachat Instruments, USA). Blank samples (i.e. natural seawater) were measured for correction of excretion rate. Scope for growth was calculated according to equation (2) [ref.^41^]:

2 $${\rm{SfG}}={\rm{AR}}-{\rm{RR}}-{\rm{ER}}$$

where SfG is scope for growth (J ind^−1^ hr^−1^); AR is absorption rate; RR is respiration rate; ER is excretion rate. Conversion factors of 14.14 J mg O_2_^−1^ and 0.025 J µg NH_4_^−1^ were used to convert respiration rate and excretion rate, respectively, into energy equivalent^43^.

### Body condition and survival of gastropods

Total weight of each individual was measured before and after the exposure experiment using an electronic balance to indicate the change in biomass. Mortality was checked daily and dead individuals were removed from the aquarium. Following the aforementioned measurements, all individuals were dissected to obtain the flesh weight which was further separated into organ weight and foot weight. Before the exposure experiment, 20 additional individuals which were collected at the same time as the experimental animals were dissected and weighed to obtain the initial organ weight to flesh weight ratio (i.e. body condition) so that the change in this ratio could be estimated^8^. All the weights were measured on a fresh weight basis.

### Statistical analysis

Two-way permutational analysis of variance (PEMANOVA) was applied to examine the effects of *p*CO_2_ and temperature on ingestion rate, assimilation efficiency, absorption rate, respiration rate, excretion rate, scope for growth, total weight, organ weight to flesh weight ratio and mortality using software PRIMER 6 with PERMANOVA + add-on.

## Electronic supplementary material

Supplementary Information
