# Supplementary material for: Heatwaves diminish the survival of a subtidal gastropod through reduction in energy budget and depletion of energy reserves
Source: Sci Rep. 2017 Dec 15;7:17688. doi: 10.1038/s41598-017-16341-1 (PMC5732251; doi:10.1038/s41598-017-16341-1)
Supplement: Supplementary file 1 — Supplementary Information [file 41598_2017_16341_MOESM1_ESM.pdf]

Supplementary Information

Heatwaves diminish the survival of a subtidal gastropod through reduction in energy budget and depletion of energy reserves

Jonathan Y.S. Leung*[1], Sean D. Connell[1], Bayden D. Russell[1,2]

[1]*Southern Seas Ecology Laboratories, The Environment Institute, School of Biological Sciences, The University of Adelaide, South Australia, Australia*

[2]*The Swire Institute of Marine Science and School of Biological Sciences, The University of Hong Kong, Hong Kong SAR, China*

*Corresponding author: jonathan_0919@hotmail.com (Jonathan Y.S. Leung)

Table S1. PERMANOVA table showing the effects of pCO_2 and temperature on ingestion rate, assimilation efficiency, absorption rate, respiration rate, excretion rate, scope for growth, total weight, organ weight to flesh weight ratio and mortality. The bold letters indicate significant difference ($p < 0.05$).

	Mean square	Pseudo-F	*p*	Pairwise comparison
Ingestion rate				
pCO_2	8.53×10^{-3}	2.45	0.156	
Temperature	5.65×10^{-2}	16.2	**0.004**	21°C < 24°C
pCO_2 × Temperature	8.01×10^{-3}	2.30	0.168	
Assimilation efficiency				
pCO_2	295	1.19	0.312	
Temperature	87.1	0.351	0.572	
pCO_2 × Temperature	218	0.877	0.380	
Absorption rate				
pCO_2	0.251	4.23	0.074	
Temperature	1.61	27.2	**0.001**	21°C < 24°C
pCO_2 × Temperature	0.173	2.92	0.126	
Respiration rate				
pCO_2	1.51×10^{-4}	4.44×10^{-2}	0.843	Within 21°C: N.S.
				Within 24°C: N.S.
Temperature	1.14×10^{-2}	3.36	0.101	Within 400 ppm: N.S.
				Within 1000 ppm: 21°C > 24°C
pCO_2 × Temperature	1.83×10^{-2}	5.37	**0.049**	
Excretion rate				
pCO_2	7.07×10^{-4}	0.199	0.668	
Temperature	6.91×10^{-3}	1.94	0.201	
pCO_2 × Temperature	1.84×10^{-6}	5.16×10^{-4}	0.982	
Scope for growth				

pCO_2	0.213	3.63	0.093	
Temperature	1.17	19.9	**0.002**	21°C < 24°C
pCO_2 × Temperature	0.302	5.15	0.053	
Total weight				
pCO_2	1.24×10^{-3}	2.05	0.158	
Temperature	7.00×10^{-3}	11.6	**0.001**	21°C > 24°C
pCO_2 × Temperature	7.77×10^{-5}	0.128	0.722	
Organ weight to flesh weight ratio				
pCO_2	7.66×10^{-5}	3.92×10^{-2}	0.844	
Temperature	1.78×10^{-2}	9.11	**0.004**	21°C < 24°C
pCO_2 × Temperature	4.19×10^{-3}	2.14	0.150	
Mortality in Week 8				
pCO_2	164.6	0.533	0.486	
Temperature	9259	30.0	**0.001**	21°C < 24°C
pCO_2 × Temperature	41.2	0.133	0.725	

Table S2. Seawater carbonate chemistry parameters in the aquaria during the 8-week experimental period under different treatment conditions. Temperature and pH of seawater were daily checked using a pH/temperature meter (HI 98128, HANNA Instruments, Germany), calibrated using NBS buffers. Salinity and total alkalinity were weekly measured using a hand-held refractometer and potentiometric titrator (888 Titrando, Metrohm, Switzerland), respectively. The pCO_2, dissolved inorganic carbon (DIC) and saturation states of calcite (Ω_{cal}) and aragonite (Ω_{ara}) were calculated using the CO2SYS program[1], with dissociation constants from Mehrbach et al.[2] refitted by Dickson and Millerro[3].

	Measured parameters				Calculated parameters			
Treatment	Temperature (°C)	pH (NBS scale)	Salinity (ppt)	Total alkalinity (μmol kg^{-1})	pCO_2 (ppm)	DIC (μmol kg^{-1})	Ω_{cal}	Ω_{ara}
21°C, 400 ppm	20.9 ± 0.02	8.13 ± 0.003	35.1 ± 0.09	2427 ± 11.9	476 ± 5.12	2175 ± 10.6	4.44 ± 0.04	2.89 ± 0.03
21°C, 1000 ppm	20.9 ± 0.02	7.82 ± 0.004	35.1 ± 0.10	2384 ± 17.5	1054 ± 19.0	2275 ± 17.2	2.36 ± 0.04	1.54 ± 0.02
24°C, 400 ppm	24.0 ± 0.02	8.13 ± 0.004	35.2 ± 0.09	2385 ± 15.4	474 ± 8.15	2114 ± 14.5	4.74 ± 0.06	3.12 ± 0.04
24°C, 1000 ppm	23.9 ± 0.02	7.83 ± 0.005	35.2 ± 0.09	2383 ± 20.1	1051 ± 23.0	2256 ± 19.2	2.64 ± 0.06	1.73 ± 0.04

References

1. Pierrot, D., Lewis, E. & Wallace, D.W.R. *MS Excel Program Developed for CO_2 System Calculations*. ORNL/CDIAC-105a. Carbon Dioxide Information Analysis Center, Oak Ridge National Laboratory, U.S. Department of Energy, Oak Ridge, Tennessee (2006).

2. Mehrbach, C., Culberso, C.H., Hawley, J.E. & Pytkowic, R.M. Measurement of apparent dissociation-constants of carbonic-acid in seawater at atmospheric-pressure. *Limnol. Oceanogr.* **18**, 897−907 (1973).

3. Dickson, A.G. & Millero, F.J. A comparison of the equilibrium-constants for the dissociation of carbonic-acid in seawater media. *Deep Sea Res. A* **34**, 1733−1743 (1987).
